# Supplementary material for: Patterns of Intron Gain and Loss in Fungi
Source: PLoS Biol. 2004 Nov 30;2(12):e422. doi: 10.1371/journal.pbio.0020422 (PMC532390; doi:10.1371/journal.pbio.0020422)
Supplement: Table S1 — Also available at http://genes.mit.edu/NielsenEtAl/. (4.3 MB ZIP). [file pbio.0020422.st001.zip › NielsenEtAl/html/1225.html]

AN2282.1.NCU00195.1.MG04927.1.FG04217.1


```
 CLUSTAL W (1.82) Multiple Sequence Alignments - Introns Inserted


Sequence 1: MG04927.1	496 aa
Sequence 2: FG04217.1	500 aa
Sequence 3: NCU00195.1	635 aa
Sequence 4: AN2282.1	503 aa
Alignment Length: 638 aa
Number Identitical Residues: 191 aa
Alignment Score (without introns) 11244


MG04927.1 	------------------------------------------------------------
NCU00195.1	MVNGGGDTVDPGHHRPSTGGAPKCVRSLCHTHPIIREPSRRPCEMEHFERRGLTPEILGQ
FG04217.1 	------------------------------------------------------------
AN2282.1  	------------------------------------------------------------
          	                                                            

MG04927.1 	--------------------------------------------------~---------
NCU00195.1	SCTHVCTMPDATIISALMHCTDPDRSASAVSVRSSASSIHVDTASDPYPS1GCCLGTRSC
FG04217.1 	--------------------------------------------------~---------
AN2282.1  	--------------------------------------------------~---------
          	                                                            

MG04927.1 	----------M~GS~APIDGSGNVAVDSERLSNEKG-----SSPAAAGHHDEVQCPPHTT
NCU00195.1	KTRIMSDIKDL1SL~EEKTHHDPSGFSSSDKGAEAGRISYDVGPNSPTTATALSPPEGTT
FG04217.1 	--------MAL~NS~REKESGNESPVSYEDPERSNT-----IGDGVVTDDVQLVCPSSTT
AN2282.1  	--------MGV~LD1RETEGSKNSRSPSREKADEVQ-----MGLESP-QPGELPLLTATT
          	          :                      .        .         :     **

MG04927.1 	ERKLLMKIDSRVIPWLCIMYLLAFLD~R~VNIANANVFGLSDELHLDG-TLYNNALVVFF
NCU00195.1	EAAIVRKIDRHIIPFIIFLYLLAFLD~R~VNIGNARSFGLEADLGLEK-VEYNTALTIFF
FG04217.1 	DSKLMARIDFHVIPFLCIMYLLAFLD1R~VNIANADVFGLSEELGLEK-TEYNTALVVFF
AN2282.1  	ERKLMAKVDWHVVPCLCIMYLLAFLD~R2VNISNAAALGLKDDLNIAEGTKYNTALTIFF
          	:  :: ::* :::* : ::******* * ***.**  :**. :* :  .. **.**.:**

MG04927.1 	VPYILFEIPSNILVKRFRPHVWL~SINMGGFGLVTLAQGFVTSYGGLVTTRFFLG~VFES
NCU00195.1	VPYIILEIPGNILMKKFSPRVWL~SICCVGFGLVSIFQGLVQNYGGLLATRFFLG~VFEC
FG04217.1 	VPYILFEIPSNILLKRFKPHVWL~SLNMFLFGLTTLLQGFVQNYSGLIATRFFLG~LFET
AN2282.1  	VPYVLFEIPSNILLKKLRPHVWL1SLCMFTFGVVTICQGLVTNWGGLMTTRWFLG1MSET
          	***:::***.***:*:: *:*** *:    **:.:: **:* .:.**::**:*** : * 

MG04927.1 	VMFPAS~FYLIGMWYKRSEAQKRYSFFFSSTTLAGAFGGLIAAGIGKMAGARGYAGWRWI
NCU00195.1	SMFPGC~FFLLASWYKRADAQKRYSLFFSSTSLAGAFGGLLASAIGKMDRLQGYRGWRWI
FG04217.1 	GMFPGA1FYLIGMWYRRHEAQRRYSFFFNSTTLAGAFGGLLAAAIGKMSGVRGYAGWRWI
AN2282.1  	GMFPGC1FYLLGMWYKRSEAQKRFSFFFSSTSLAGAFGGVLASGLGKMHGVRNLAGWRWV
          	 ***.. *:*:. **:* :**:*:*:**.**:*******::*:.:***   :.  ****:

MG04927.1 	FIIEGALTVFVSFFFYFLLPDFPEEAKWLTEEERQFVTARLRVDQGNSAVEKKITLRDVG
NCU00195.1	FIVEGCVTFLAGVLFFFTFPAFPEEARFLKPAEREYVQAMLQADHGHSAAERKITLRDVG
FG04217.1 	FILEGGLTVLVSFFFYFLLPNFPEQAKWLTPEEKDYVTARLRADQGHAGAERKTTFSDAL
AN2282.1  	FLIEGVLTAVVAIILFFFLPDFPEEAKWLTEEERAYMRAKLAQDSGEAGEDTRMGWREVL
          	*::** :* ....:::* :* ***:*::*.  *: :: * *  * *.:. : :    :. 

MG04927.1 	RVFKDY~KVIVAGFMYFGLIVPA1YG~YAYFAPSII0ASYGYSP~IQTQLHSVPPWVAAF
NCU00195.1	KALTDH~RIWLGGIMYLGLIVPA~YS~YAYFSPTII~GSYKYSA~IQTQLHSVPPWAAAF
FG04217.1 	NVLKDY~KIIIGGFMYFGLIVPA~YG~YAYFAPGII~KTYGYSP~IQTQLYSVPPWAAAF
AN2282.1  	AVFKDY1KIFIGGLMYFGQIVTA~YG1YAYFAPTII~QSYGYGP1IQTQLYSIPPWAGAF
          	 .:.*: :: :.*:**:* **.* *. ****:* **  :* *.. *****:*:***..**

MG04927.1 	GMSMLFAVASDRMRHRWGFAVFAILVAIAGFSILLGVHN--NTDVQYAGLFLIAMGAYTA
NCU00195.1	GFAMVTAVASDYLRHRFMFAILGIAVAIAGFAILMTVHDPAHVNVQYGGLFLVAMGAYSA
FG04217.1 	AFSLCVAFASDKMRHRACFAIFSILVAIAGFATLLSVHD--NHKVQYGALFLVTMGAYTA
AN2282.1  	GFSMCIAYISDKFRHRFAFTIIPMLISMAGLGILMNVHD--NRDVQYGALFLVTAGCYSA
          	.:::  *  ** :***  *::: : :::**:. *: **:  : .***..***:: *.*:*

MG04927.1 	MPIIVCWFNMNLGGHHRRAVGSAWQVGFGNI~GGIIAVYAFLKRDAP-LYIPGYSICIAF
NCU00195.1	MPIIVCWFNMNLGGHHRKAIGTAWQIAFGNI~GGIIATYSFVASDAKNHYRMGYAICVSF
FG04217.1 	MPIIVCWFNMNLGGHRRRSIGSAWQVGFGNI~GGIIAVYSFLQKDAP-KFIRGYSICIAF
AN2282.1  	MPVVVCWYAMNLGGHRRRSVGTAWQVGFGNI1GGIIATYSFLAKDRTNNYYTGFSIGISF
          	**::***: ******:*:::*:***:.**** *****.*:*:  *  . :  *::* ::*

MG04927.1 	TTLSIIACTIYGFACMSANKKRARLTQSG----TVEENSDLGDLSPSYRYLL
NCU00195.1	TCLSALACLLYAISVTMENKRRAKMQGDGG--LTEEEKADLGDLNPEFRYML
FG04217.1 	TILSIIACITYGAVCLYENRKRAANPPERE--LTDDEKAELGDNAPTYRYLL
AN2282.1  	LAFSCACCCVYFAAVWHQNQKKDRILASGDPSYLEEDEAVLGDQATKFRYAY
          	  :*  .*  *       *:::     .  .:   :::: ***  . :**
```
